# Canine circovirus: an emerging virus of dogs and wild canids

**DOI:** 10.1186/s13620-025-00290-7

**Published:** 2025-02-08

**Authors:** Farzad Beikpour, Arash Letafati, Zahra Ahmadi Fakhr, Nicoal Decaro, Sayed-Hamidreza Mozhgani

**Affiliations:** 1 Department of Pediatrics, Washington University School of Medicine, St. Louis, MO USA; 2 Department of Virology, Faculty of Public Health, Tehran University of Medical Sciences, Tehran, Iran; 3 Research Center for Clinical Virology, Tehran University of Medical Science, Tehran, Iran; 4 Department of Biology, College of Convergent Sciences and Technologies, Science and Research Branch, Islamic Azad University, Tehran, Iran; 5 Department of Veterinary Medicine, University of Bari Aldo Moro, 70010 Valenzano, Italy; 6 Department of Microbiology and Virology, Alborz University of Medical Science, Alborz, Iran

**Keywords:** CanineCV, Circovirus, Canids, Animals, Co-infection

## Abstract

Canine Circovirus (CanineCV) is an emerging viral pathogen affecting dogs and wild canids worldwide. Belonging to the Circoviridae family, CanineCV exhibits genetic variability and has been associated with various clinical manifestations, including gastroenteritis, respiratory symptoms, and neurological disorders. While its prevalence is notable, gaps persist in understanding its pathogenicity and evolutionary origins. CanineCV often co-infects with other canine viruses, complicating diagnosis and treatment. Prevention strategies are hindered by the lack of targeted vaccines and the virus's resilience in the environment. This review paper summarizes the current knowledge on CanineCV and discusses the virus's genetic features and taxonomy, epidemiology in both domestic and wild populations, clinical manifestations, diagnosis methods, and prevention strategies. Additionally, the article highlights gaps in knowledge regarding CanineCV's pathogenicity, evolutionary aspects, and potential zoonotic transmission risks. Overall, it underscores the importance of further research to better understand and mitigate the impact of CanineCV on canine health and public health.

## Introduction

The evolution driving the emergence of novel viruses is thought to be related to interactions within the agent-host-environment triangle and other known and unknown factors [1]. In recent decades, the optimization of advanced and accurate diagnostic techniques has allowed for the discovery of novel viruses in humans and animals. The circular, Rep-encoding ssDNA (CRESS-DNA) viruses are a large group of viruses with circular single-strand DNA and replicase-associated protein (*rep)* [2].

*Circoviridae* is a relatively large viral family within the large group of CRESS DNA viruses, encompassing important animal viral pathogens [2]. The *Circoviridae* family is divided into two genera, namely *Circovirus* and *Cyclovirus*, retrieved from a broad range of hosts such as mammals [3], birds [4], fish [5], insects [6], plants, and from the environment [2, 7]. The number of viral species identified within both genera has recently soared. Yet, the natural reservoirs of the majority of these viruses remain unknown [3, 7]. One of the recently discovered species is Canine Circovirus (CanineCV), which was first reported in 2012 in the USA [8]. Since then, there have been several reports on CanineCV in both domestic and wild carnivorous populations worldwide. Interestingly, virus circulation has been backdated at least to 1996 in the population of the arctic fox, as researchers found the CanineCV genome in the samples collected before 2012 [9, 10].

Although the number of countries and animal species in which CanineCV has been detected significantly increased, there is still a gap in knowledge about the virus’ pathogenic role and evolutionary aspects [11, 12]. So far, CanineCV has been frequently found in co-infections with other canine viruses [10, 13, 14].

CanineCV has been associated with fatal and non-fatal hemorrhagic enteritis and diarrhea [15, 16], vasculitis and hemorrhage [17], and respiratory symptoms [18]. However, the virus has been found in healthy dogs as well [19], Also, the virus is reported to be more prevalent in animals that show respiratory or diarrhea symptoms, which indicates its association with animal diseases [20]. A CanineCV-like virus has also been detected in brain samples of foxes that displayed unexplained meningoencephalitis and neurologic signs [21].

Gastroenteritis is a multi-etiology illness, with viruses being regarded as the most common causative agents in dogs, as they are detected in 40–60% of diarrheal samples, resulting in high morbidity and mortality, particularly in unprotected animals that have not been vaccinated [22, 23]. Although, there have been studies suggesting the presence of virus and disease being associated [24, 25], some other studies report no correlation [13, 16]. The idea linking diarrhea to CanineCV is based on insights gained from the pathogenic behavior of other similar viruses, like porcine circovirus (PCV) [23]. Studies have indicated that in PCV, certain proteins are associated with virulence and disease severity, while similar roles for CanineCV proteins are still being investigated. Moreover, CanineCV infections often occur alongside other pathogens, particularly Canine Parvovirus type 2 (CPV-2), complicating the assessment of CanineCV's specific impact on gastrointestinal diseases like diarrhea [26]. In a molecular study on dogs with gastrointestinal problems, the PCR results revealed that out of 127 adult dogs with gastrointestinal problems, 8 tested positive only for CanineCV, and 7 had dual infection with Astrovirus, which can induce disease in multiple animal species and has been reported as the leading cause of diarrhea in dogs in the referenced study in Turkey [27, 28].

The transmission of the virus is thought to occur through direct contact with infected animals and/or contaminated secretions or excretions, as the virus is durable in the environment, and because an infected dog can shed the virus more than a year post-infection. Yet, there is no report of dog-to-human transmission of CanineCV. Researchers continue to investigate the complete epidemiology and pathogenic mechanisms of CanineCV. As their studies advance, they may uncover additional insights into its behavior in canine populations; however, current evidence indicates that there is no risk of transmission to humans [29].

## Virus genetic features and taxonomy

**Fig. 1:**
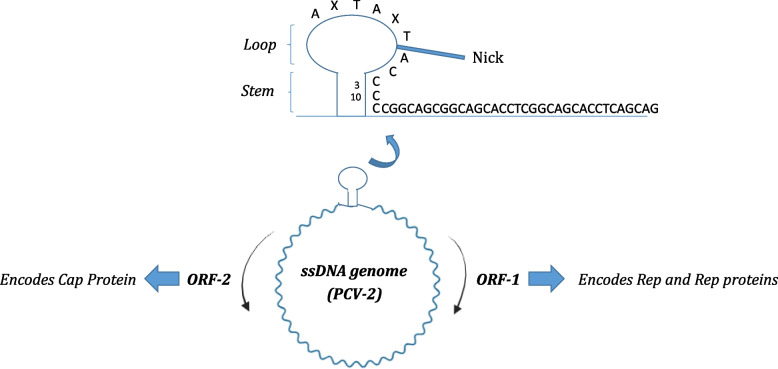
Genomic structure of CanineCV

With an almost 20-nm diameter in size, the genus *circovirus* comprises the smallest self-replicating ssDNA viruses of mammals that are covalently icosahedral and non-enveloped [11, 19, 30]. CanineCV has an approximately 2 kb circular genome and possesses two inversely organized open reading frames (ORFs) encoding Rep and Cap proteins (Fig. 1), which are 303 and 270 amino acids in length, respectively. While the Rep gene seems to be more conserved [14, 30], the cap ORF is more heterogeneous, as observed in other enteric viruses [31]. Between the two ORFs, there is a conserved 9-nucleotide (TAGTATTAC) stretch, the origin of replication, with a stem-loop stable structure that initiates the rolling-circle replication process [8]. Recently, an additional ORF which is ORF3, has been proposed to be anti-directionally located within the ORF1 region, but its function needs to be decoded [18, 31]. The virus is thought to use a circular, double-stranded replicative form (RF) transitional DNA by recruiting the cellular DdDp enzyme during the S phase of the cell cycle to replicate its genomes. The RF subsequently acts as a template for the production of more viral ssDNAs to proliferate virus particles, and the DNA replication process is most likely accomplished through the rolling circle replication (RCR) procedure [30].

Circoviruses can cross the species barrier and adopt a new host, as these viruses show high genetic plasticity and flexibility [11, 19]. This feature may have derived from genetic mutations and/or genomic re-combinations, as the genetic analysis already revealed different sites across the CanineCV can undergo natural selection to get single-point mutations and/or recombination events [18, 31, 32] with purifying selection being the dominant evolutionary pressure acting on the CanineCV genome [20, 32].

Studies have reported the detection of four potential recombination events in the two ORF genes, which constitute the complete genome of CanineCV, as well as other parts of the genome. These events included intergenotype and intragenotype recombination, and the major and minor parents were from China, Europe, and North America, which highlights the importance of close monitoring of CanineCV genomic recombination [18, 20, 31, 33, 34].

## Epidemiology

### CanineCV in wild carnivores

Until November 2023, CanineCV has been detected in North and South America, Europe, and Asia, spreading through more than 15 countries worldwide including Argentina [35], Brazil [36], China [12], Colombia [12], Germany [16], Italy [15], Thailand [18], Vietnam [31].

The detection rate ranges between 1 and 30.42% [9, 27, 37]. Although the first detection of CanineCV was reported from the USA [8], the distribution of the virus in the U.S. is not yet known, but dogs infected with circovirus have been reported in California, and circovirus may be associated with the recent illness and death of dogs in Ohio. In some other cases virus was reported to be alone or in combination with other viruses in sick dogs within the country [17, 29, 38].

CanineCV has been found in 1.34% of healthy dog blood samples in Brazil [36]. A full genome characterization of a Brazilian virus has been reported in the stools of a dog with vomiting and signs of intermittent hemorrhagic gastroenteritis, fed with raw pork [39]. Recently, a co-infection case of CanineCV and CDV has been reported in Brazil from the lung and intestinal tissues of a dog [40]. CanineCV has been discovered in several organs, including the lung, kidney, lymph nodes, pancreas, spleen, liver, and large intestine, of sick dogs in an episode of fatal gastroenteritis in Argentina [35]. The virus was detected in 16.6% CPV-2 positive dogs and, on phylogenetic analysis, the CanineCV from South America showed a close evolutionary correlation with European strains [37].

In Europe, there have been several reports on CCV in dogs. The first report of the virus described a fatal outbreak of enteritis in young dogs from Italy [15]. In a 2015 study, 3.8% of the internal organs of dogs from the central part of Italy tested positive for CanineCV. Interestingly, all these CanineCV-positive samples were also positive for CDV or CPV-2 [41]. In a 2017 case–control study from Italy, CanineCV was the second most prevalent virus in the studied group, even if a direct correlation between CanineCV and diarrhea was ruled out [13]. These results were consistent with the findings of a 2019 study in Germany [16]. Also, screening of 95 enteric samples collected between 1995–2017 in Italy dates back the circulation of CanineCV to 2009 [10].

The majority of CanineCV reports in Asia come from studies in China, Thailand, Vietnam, and Taiwan. In China, in one study, more than one-third of the screened diarrheal dogs were positive for CanineCV [24], which was higher than other reports from China that Niu et al., could detect the virus in 13% of their sampled dogs. They also reported co-infection of the virus with CPV-2 and found that two different strains of CanineCV were present in the same sample [12]. A large Chinese epidemiological study screened more than 1200 dog samples. CanineCV was not detected in 4 provinces, while in Guangxi province, about 9% of dogs were CanineCV-positive and most dogs were clinically healthy [34]. A study in Thailand in dogs with respiratory signs identified putative recombinant viruses [18].

With a detection rate of 0.013, another recent study proposed evidence for CanineCV’s significant association with canine respiratory disease, suggesting that dogs with such symptoms were 5.6 times more likely to be positive for CanineCV compared to healthy dogs [42]. The highest rate (20%) of detection of CanineCV has been reported in Vietnam, and genome sequencing showed two different genotypes (CanineCV-1 and -3) in the country [31]. In west Asia and the Middle East, there are only three reports published on the virus’s circulation, two of which have been documented in Iran. While the first report from the region comes from Turkey, showing a 6% prevalence [27]. The most recent papers from Iran revealed a higher rate of infectivity; 9% of healthy kennel dogs were positive for a novel genotype of the virus (CanineCV-6) in different provinces in Iran [19]. Also, Faraji et. al. reported more than 25% of their CPV-positive dogs were co-infected with CanineCV resulting in worsening the dogs’ health status [14].

Although CanineCV has been regarded as a canine virus, it has also been found in other animals, including cows and domestic cats [43], with a recent study reporting a 2.75% prevalence of CanineCV in cats [20]. There is a recognized need for further research to clarify the modes of transmission and the impact of domestic animals on wild canid health. Current findings indicate that while there are correlations between domestic dog populations and pathogen presence in wild canids, definitive conclusions about infection pathways require more comprehensive studies involving direct observation and molecular diagnostics [11, 44].

In foxes, CanineCV and genetically related viruses have been reported repeatedly. The first report was published in 2015, when a fox circovirus was detected in brain and serum samples from foxes in the UK. The foxes showed unexplained neurologic and meningoencephalitis symptoms [21]. In 2015, another survey in Italy reported CanineCV in 26% of internal organ samples of wolves and almost 14% of badgers, but not in foxes [41]. A more recent surveillance study in Italy reports that 18%, 50%, and 0% of badgers, wolves, and foxes, respectively, are positive for CanineCV [45]. Likewise, internal organ samples from Italian wolves have been shown to be infected with CanineCV at an even higher rate. CanineCV was detected in more than 47% of tested wolves, mostly coinfected with CPV [46]. The sequences determined in wolves in this study were clustered together with five CanineCV sequences obtained from another study on samples collected from Chinese Harbin City pet dogs. This indicates CanineCV’s potential for cross-species transmission between dog and wolf, despite the unavailability of direct evidence to support it. Moreover, the long-term evolutionary relationship between circovirus and canid host is suggested by the considerable geographical distance between the two populations [20, 32, 46].

**Fig. 2:**
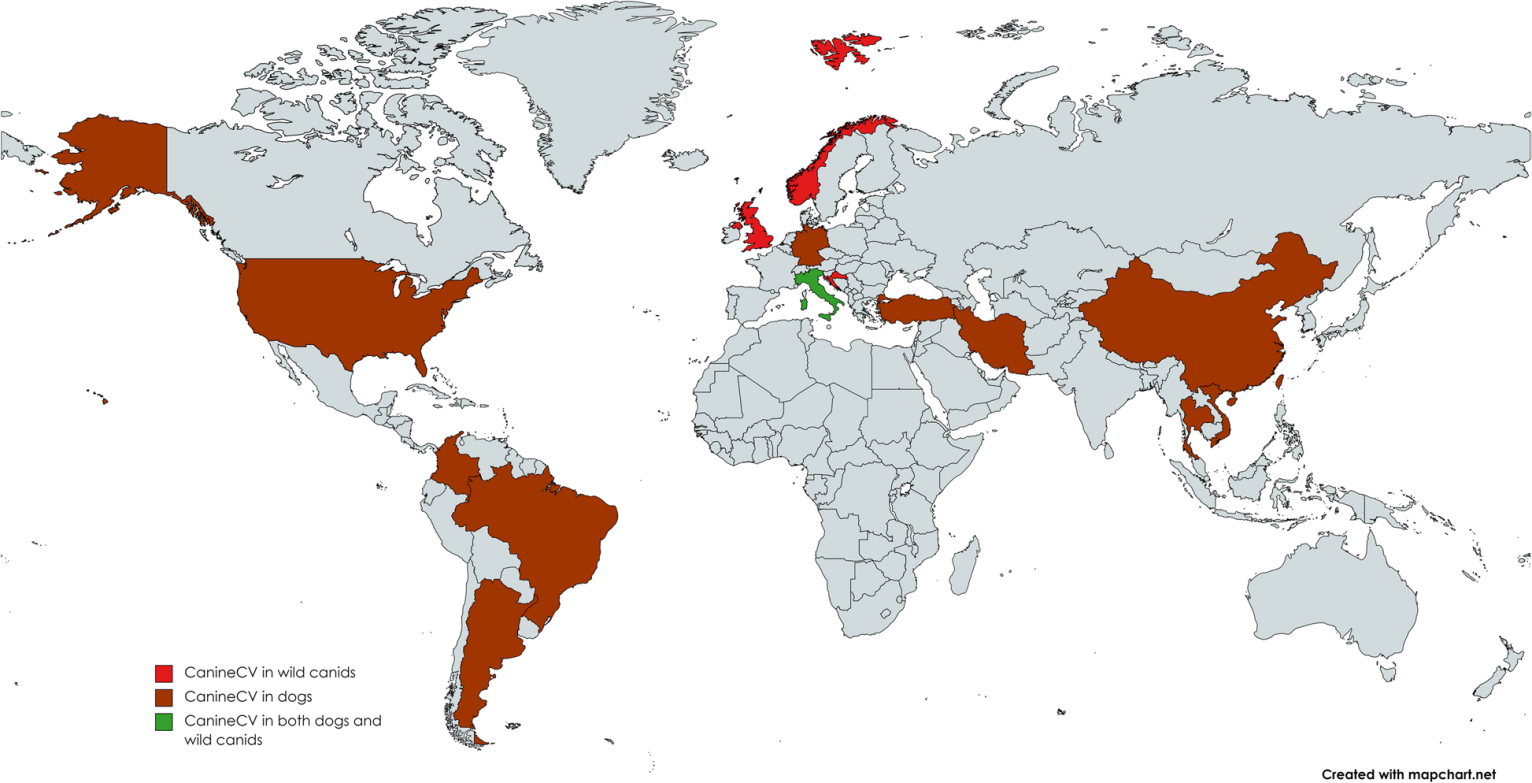
In this figure, the reports of CanineCV mentioned in the epidemiology section can be seen according to Wild canids, dogs and both (Created by mapchart.net)

Franzo et al., reported CanineCV in 2–5% of the foxes from two distinct regions in northern Italy [11]. A CanineCV strain in fox was phylogenetically more similar to dog strains than to fox strains, highlighting a potential transmission between these two species [47]. Evaluation of the fecal virome of red foxes in suburban areas in Croatia has revealed that CanineCV was the most prevalent virus among juvenile foxes [48]. In another study in Norway, CanineCV was detected in both Arctic and red foxes. Also, the virus was detected in a sample from 1996 [9]. Figure 2 depicted reported animals divided by country infected with canineCV.

CanineCV has been recently proposed to comprise 6 genotypes that are well separated in phylogenetic analysis: CanineCV-1, reported mainly in the USA, Europe, and Asia [20], includes strains detected in dogs, wolves, and badgers; CanineCV-2, 3, and 4 include strains circulating in east and southeast Asia; CanineCV-5, detected in Europe and North America and is related to wild canids, mostly foxes, CanineCV-6 comprises strains recently reported from Iran [9, 12, 19, 31]. Only CanineCV strains that were identified in Iran or China had formed genotypes 2, 3, and 6 [20, 32]. The sequence identity in all these strains was reported to be shared by 86 to 100 percent [20]. Phylogenetic and comparative analyses have proposed the bat circovirus as an ancestor of CanineCV [35]. Accordingly, it is speculated that the virus has emerged from wild animal populations in domestic dogs, resembling the scenario proposed for Canine Parvovirus-2 (CPV-2) [9]. So far, the virus has never been cultured in vitro or in vivo.

## Tropism and pathophysiology

Attempts to evaluate host-virus interactions in a laboratory animal model so far have been unsuccessful [15, 31, 39]. Recently, through genetic modification of the virus, it has been successfully rescued in Feline kidney (F81) cells [43]. CanineCV is thought to be associated with gastroenteritis alone or in combination with other enteric viruses, such as CPV. Yet, the virus has been detected in other organs like the liver [17], spleen [9], brain [21], lymph nodes [41], serum [8], and tonsils [18]. Moreover, several studies have documented co-infection of CanineCV with other viruses such as CPV and CDV [10, 14, 16, 40]. Importantly, recent studies have shown that CanineCV can suppress immune responses by blocking type 1 interferon promotor [43]. The synergistic impact of circoviruses and parvoviruses has already been reported in other mammals [37]. CanineCV and CPV co-infection has been proven to worsen the clinical situation of the infected animals [14, 43]. CPV infection results in necrosis of crypt epithelial cells and lymphocytes, and subsequent proliferation of epithelial cells and lymphoblasts provides relevant target cells for CanineCV replication [10]. Also, it is hypothesized that co-infection may even impact the infected dog’s immune response and viral clearance [29].

It has been described that CanineCV could easily be multiplied in the lymphoid tissues of dogs that had previously developed pathology as a result of parvovirus infection [29]. Unlike these studies that report highly frequent infection in cases with CPV-2, samples that were not previously diagnosed with such infection were investigated in a recent study that demonstrated a higher-than-expected frequency of the CanineCV in diarrheic animals with no CPV-2 coinfection, which exposes the sub diagnosis of the virus [49].

**Fig. 3:**
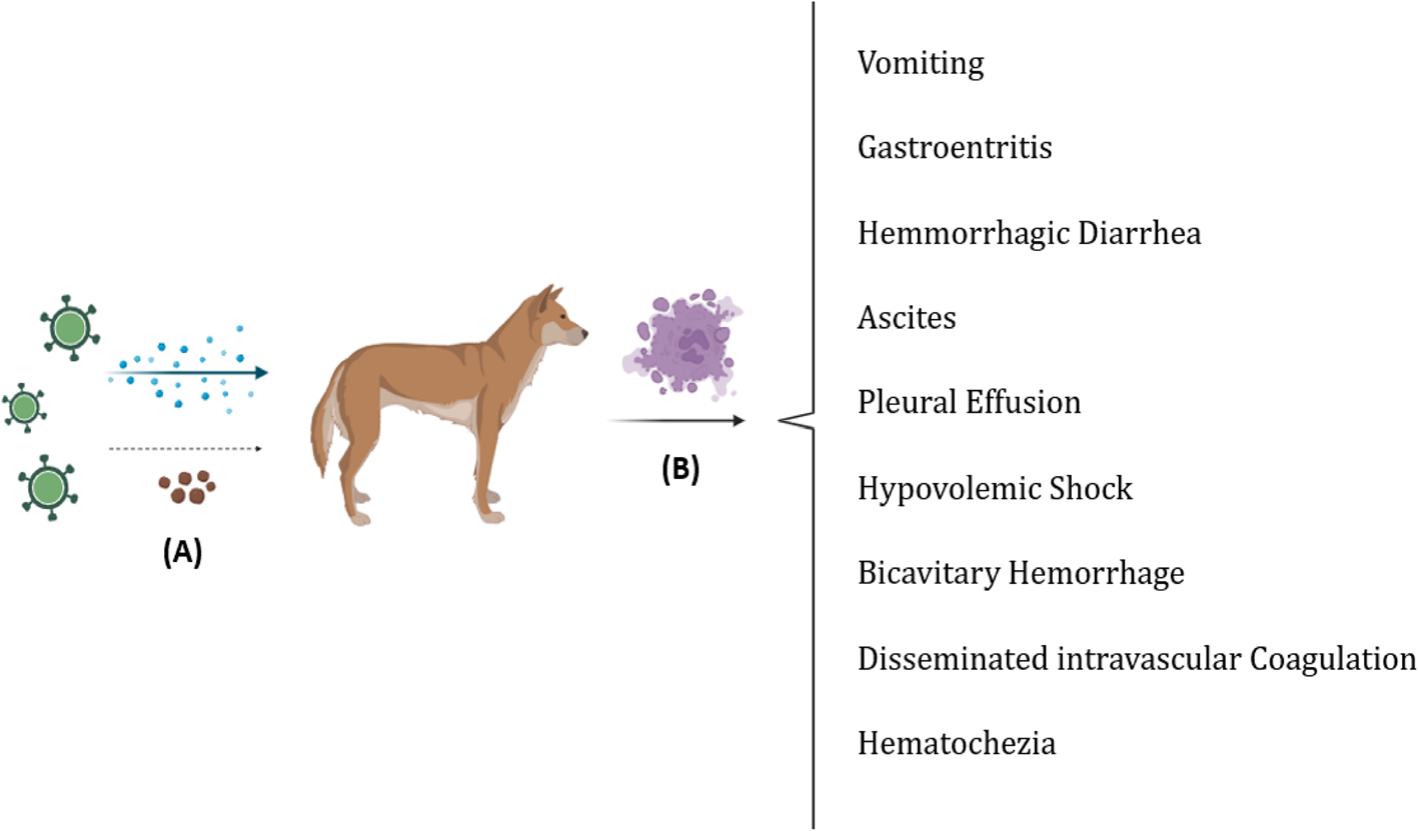
Following the inhalation of contaminated respiratory droplets and feces from an infected animal (**A**), viral replication occurs in crypt and lymphocyte cells. Subsequent to this event and the cessation of type I interferon production by the virus, we observe necrosis of intestinal crypt cells (**B**) in the animal, followed by clinical symptoms associated with the virus (Figure is created with BioRender)

It has also been described that the position and degree of impacted vessels differ amongst CanineCV-infected dogs. In one study, the majority of dogs displayed anomalies in the gut and kidneys. Lymphocytic infiltrates into lymphoid organs were found in most cases, similar to circovirus infection in other animals. In dogs infected with CanineCV, however, neither viral inclusions nor multinucleate giant cells, which are significant histologic hallmarks of PCV-2 infection, were observed by routine histology. CanineCV is invariably disseminated in the cytoplasm of macrophages and monocytes inside the lymphoid tissues of infected dogs, according to in situ hybridization (ISH) investigations, as observed for porcine circovirus [11, 34, 50]. Accordingly, some studies show that CanineCV may be associated with immunosuppression and lymphoid depletion [24, 44]. Also, dogs with circovirus infection may develop hematochezia, hemorrhagic diarrhea, and increasing vomiting, and possible conditions include ascites, pleural effusion, hypovolemic shock, bicavitary hemorrhage, and disseminated intravascular coagulation (Fig. 3). Therefore, Circovirus should be investigated in cases of unusual vasculitis in dogs [17].

## Diagnosis, prevention and control

CanineCV diagnosis mostly relies on molecular detection using PCR or qPCR. Two common samples in this area are the fecal sample (especially when the dog has symptoms of diarrhea) and the blood sample in cases where there are symptoms such as fever. In infected animals, thrombocytopenia and neutropenia are observed in infected animals [17].

In a study focused on molecular detection of the virus, the authors developed a hydrolysis probe-based real-time PCR assay for the detection of CanineCV. The assay demonstrated a detection limit of 8.42 × 10^1^ copies/μL, making it approximately 1000-fold more sensitive than traditional PCR, which had a detection limit of 8.42 × 10^4^ copies/μL. The real-time PCR method showed high specificity, sensitivity, and repeatability, with no cross-reactivity with other pathogens. This method's enhanced sensitivity and reliability make it valuable for early-stage infection detection and epidemiological investigations of CanineCV [51].

Concomitant viral infections and differentiation of diagnosis in canines such as CPV, CDV, CCV, which have nearly similar clinical symptoms are significant due to their implications for canine health and disease management. These infections often occur together, leading to severe clinical outcomes, especially in domestic and wild dogs [12, 52]. In this case, Multiplex PCR methods have been reported to be efficient, sensitive, and specific testing tools available at a low cost for CanineCV and some other canine enteric viruses, namely CAV-2, CCoV, and CPV [53]. Moreover, a recent protocol has been developed for the detection of CanineCV antibodies using recombinant capsid enzyme-linked immunosorbent assays [17, 54].

Mixed infections involving canine viral pathogens, including Canine Distemper Virus (CDV), Canine Adenovirus (CAdV-1 and CAdV-2), and Canine Parvovirus (CPV-2), present diagnostic challenges due to overlapping clinical, histopathologic, and immunohistochemical findings. A study highlighted the broad tissue tropism of CDV, with antigens detected in epithelial cells of the respiratory, gastrointestinal, and central nervous systems. Co-infections were common, with cases of interstitial pneumonia, necrohaemorrhagic hepatitis, and parvoviral enteritis showing concurrent infections of CDV with CAdV-1, CAdV-2, and CPV-2. Immunohistochemistry (IHC) was critical for identifying specific pathogens within tissues and differentiating their contributions to disease. For example, CDV antigens were observed in areas of white matter demyelination, while CAdV-1 was localized to hepatocytes and Kupffer cells in cases of infectious canine hepatitis. CPV-2 was identified predominantly in intestinal crypts and myocardium, and Neospora caninum was detected in cases with granulomatous pneumonia and encephalitis.

The findings emphasize the necessity of comprehensive diagnostic approaches, including IHC and molecular assays, to accurately identify co-infections in canine viral diseases. In the context of canine circovirus (Canine CV), these insights underline the importance of considering mixed infections, as co-infecting pathogens could exacerbate clinical manifestations or obscure the primary role of Canine CV in disease progression [55].

Safeguarding dogs during the period when maternal immunity diminishes is deemed the most effective preventive measure. Given the lack of identified reservoirs for the virus and its potential resilience to environmental factors, additional research is warranted in this area. Also, the lack of medicine and vaccines may help the virus spread more and cause even more consequences [30, 56].

## Conclusion

Canine Circovirus (CanineCV) represents a significant emerging viral threat to canine populations worldwide, including both domestic dogs and wild canids. Through a review of the available literature, this paper has elucidated various aspects of CanineCV, including its genetic features, taxonomy, epidemiology, clinical manifestations, diagnosis, and prevention strategies. The virus's genetic variability and propensity for co-infections with other canine pathogens pose challenges for diagnosis and treatment. While CanineCV has been associated with gastroenteritis, respiratory symptoms, and neurological disorders, its precise pathogenic role and evolutionary origins remain areas of active investigation. Additionally, the potential for zoonotic transmission underscores the importance of further research to better understand CanineCV's impact on both canine and human health. Addressing knowledge gaps in CanineCV epidemiology, pathogenesis, and transmission dynamics will be crucial for developing effective prevention and control measures to mitigate its spread and impact on canine populations and public health.

## Data Availability

No datasets were generated or analysed during the current study.
